# Advances in biologging can identify nuanced energetic costs and gains in predators

**DOI:** 10.1186/s40462-024-00448-y

**Published:** 2024-01-22

**Authors:** Holly M. English, Luca Börger, Adam Kane, Simone Ciuti

**Affiliations:** 1 School of Biology and Environmental Science, University College Dublin, Belfield, Dublin, Ireland; 2 Department of Biosciences, Swansea University, Swansea, UK

**Keywords:** Predation, Energetics, Foraging, Movement ecology, Biologging, GPS, Accelerometer, Social system

## Abstract

Foraging is a key driver of animal movement patterns, with specific challenges for predators which must search for mobile prey. These patterns are increasingly impacted by global changes, principally in land use and climate. Understanding the degree of flexibility in predator foraging and social strategies is pertinent to wildlife conservation under global change, including potential top-down effects on wider ecosystems. Here we propose key future research directions to better understand foraging strategies and social flexibility in predators. In particular, rapid continued advances in biologging technology are helping to record and understand dynamic behavioural and movement responses of animals to environmental changes, and their energetic consequences. Data collection can be optimised by calibrating behavioural interpretation methods in captive settings and strategic tagging decisions within and between social groups. Importantly, many species’ social systems are increasingly being found to be more flexible than originally described in the literature, which may be more readily detectable through biologging approaches than behavioural observation. Integrating the effects of the physical landscape and biotic interactions will be key to explaining and predicting animal movements and energetic balance in a changing world.

## Introduction

**Table:** 

**Box 1. Key outstanding questions in predation energetics**		
Predation is an ecologically critical behaviour, dictating predator energy budgets with cascading effects for prey. Predation can be difficult to observe and study in the wild however, and there remain knowledge gaps which are further complicated by variation between individuals and social systems. Some key outstanding questions may be filled using developments in animal-attached technology.		
• How do environmental factors and within- and between-species interactions affect how prey are located, selected and captured, in both stable and changing habitats?		
• How can we refine detection and quantification of complex, variable predation behaviours, such as those involved in handling prey and feeding?		
• Are key predation dynamics incompletely captured by commonly used data collection strategies? For example, are intra-group interactions and hunting roles missed when few animals within a social group are tagged?		
• How do hunting dynamics change if predators and their prey are unequally affected by climate change and habitat modification?		

Animals adapt their behaviour to optimise gains and minimise losses in an environment, with energetic, ecological and evolutionary consequences [1]. Foraging is a sequence of continuous behavioural decisions made to maximise energetic gains while minimising costs in the search for food and its handling [2, 3]. Animals are faced with multiple foraging decisions, for example whether to target one prey species over another [4] or whether to forage cooperatively with conspecifics [5]. The costs associated with foraging are especially pertinent in predatory animals which must invest energy in the pursuit and handling of prey, often with risk of injury to themselves [6]. Foraging costs for predators are determined by the potential profitability of each prey item, encounter rate and handling time [7]. Whether the predator is social or solitary and the number of individuals in a cooperatively foraging group also affect individual prey selection and energy gain [8].

Foraging strategies are shaped by external factors, such as resource availability and environmental conditions [9, 10], leading to considerable variation in foraging strategies within and between individuals, social groups, populations, species and taxa [11–15]. Flexibility in foraging strategy can occur in each of these levels. Individuals may display multiple foraging strategies (i.e., switching between multiple food types which require different handling) in complex or variable environments [16], including dynamic switches regarding the tolerance of satellites by territory owners [17]. Distinct strategies may be associated with particular populations or habitats across temporal scales [18]. For example, bluegill sunfish (*Lepomis macrochirus*) modify their foraging search speed between open-water and vegetated habitats [19], and foraging trip duration and rate of chick provisioning can vary between colonies of wedge-tailed shearwaters (*Puffinus pacificus*) [20]. This variation across contexts, the difficulties associated with observing predation events, and the stochasticity inherent in food encounter rates (the role of ‘luck’ in finding food [21]), leave many open questions in our understanding of predator energetics (Box 1).

### Predation is costly

**Fig. 1:**
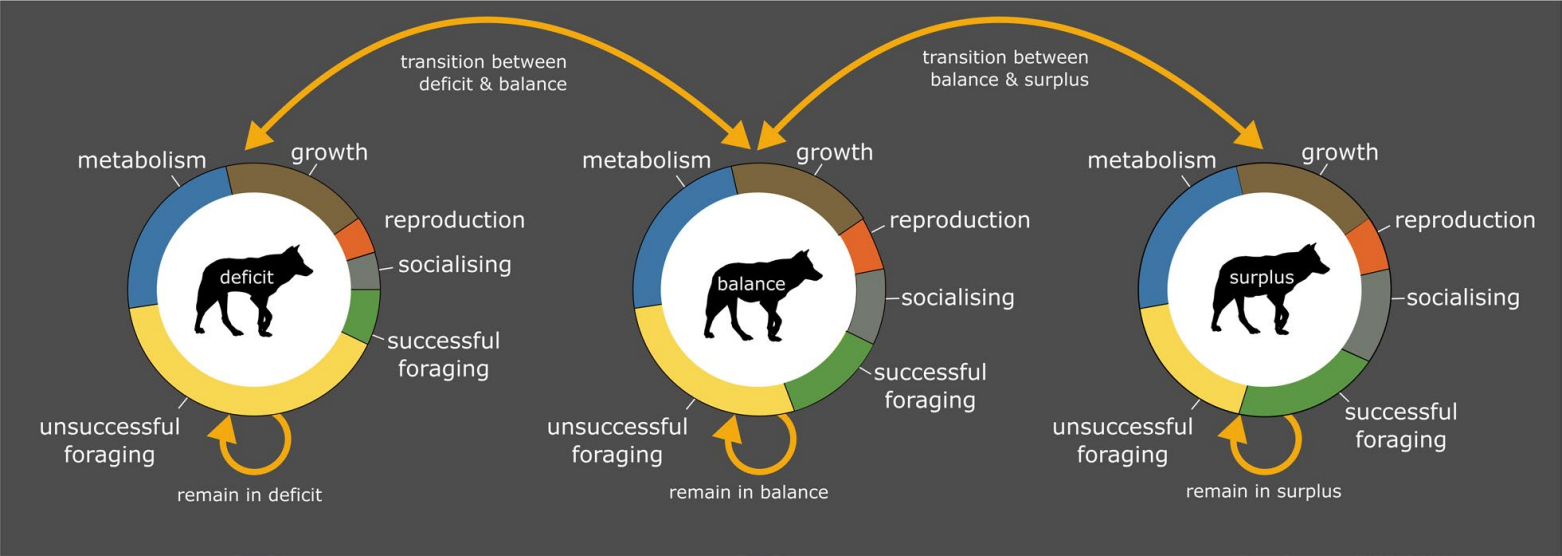
Animals can be in energy deficit, energy balance or energy surplus. The arrows here indicate that animals may remain in or transition between these states, mediated by foraging success. Animals in energy deficit incur costs which affect body condition and eventually breeding failure and death will occur if animals cannot regain energy balance. Energy balance allows normal daily functioning, while surplus energy allows investment in growth, reproduction and social behaviours

Predation typically incurs high energetic costs, either through pursuing and subduing prey, for example in large mammalian predators such as African wild dogs (*Lycaon pictus*) and lions (*Panthera leo*) [22, 23], or through shorter ambushes which require sudden bursts of energy, seen in diverse taxa including mantis shrimp [24] and snakes [25]. Hunting success is a central consideration in predation energetics. Predators must intake enough energy to account for the hunt which has just taken place, but also unsuccessful hunts since the last meal, competition e.g., through kleptoparasitism [26, 27], their basal metabolic rate, and other behaviours required for survival, growth and reproduction (Fig. 1). Meeting these diverse demands may promote flexibility in foraging behaviour, with species implementing more diverse suites of predation strategies than can easily be observed and studied using standard methods [27, 28]. This can increasingly be rectified with the use of animal-attached technology to reveal out-of-sight animal behaviours across multiple species [29, 30]. Such insights into predator energetics are valuable given the increased demands of predation compared to other foraging methods, related to locating, restraining and handling prey, which we review in full here.

### Climate and land use change may cause shifts in predator–prey dynamics

Environmental conditions can add further energetic costs to foraging [31], for example, rising temperatures may subject predators to heat stress during pursuit [32]. Prey species are subject to this pressure as well, but for many predator–prey pairs, it is unclear whether the species are equally (un)affected or whether temperature changes could shift the balance in favour of one species or the other. From the predator perspective, this could shift prey preference, with cascading ecosystem effects [33]. For marine predators, endotherms seem to have a competitive advantage over ectotherms at lower water temperatures, with consequences for species distributions [34]. In terrestrial systems, cursorial predators (which chase prey) are more likely to be adversely affected than stalk and ambush predators, due to the additional energetic costs associated with pursuing prey over large distances [35]. Though disparities in prey *versus* predator responses to rising temperatures may also work in favour of the predator, if prey become more easily exhausted under heat stress. These concepts are understudied at present, especially given the precedence of indirect climate change impacts on ecosystems. Where studies have been carried out, there is disagreement on predator–prey dynamics under rising temperatures, for example in the case of the African wild dog, where there have been contrasting findings on whether the wild dogs themselves or their prey are more impacted by heat stress associated with rising temperatures [36, 37]. These discrepancies may be partially explained by differences in prey preference across populations [32]. Assessing the energetics associated with different hunting and evasion strategies across populations is therefore a key consideration for understanding shifting predator–prey dynamics under climate change.

Land use represents another key form of global change with consequences for predator–prey dynamics, often working in tandem with climate change impacts [38]. In some cases, land use change can benefit predators by improving search efficiency as vegetation is thinned or removed [38, 39]. These dynamics can be complex, however, and vary significantly between land use types. For example, pumas (*Puma concolor*) were found to have higher body condition scores in areas of marginal anthropogenic development than in both wilderness and highly developed areas [40]. Socio-ecological phenomena must be considered as habitats are modified; land use change increases human-wildlife conflict, particularly when predators of degraded habitats target livestock [41]. Within increasingly human-dominated landscapes, some prey take advantage of carnivore avoidance of areas of high human activity, a phenomenon known as the human shield [42, 43], while others show stronger avoidance of human activity than their natural predators [44]. Understanding these complex dynamics is a priority under ongoing habitat modification and degradation, particularly given the disparity in observed species’ responses across both predators and prey.

### Energetic landscapes reveal foraging costs

Climate and land use change may cause animal populations to shift in distribution [45] with consequences for how hunting animals locate, select and subdue their prey. Shifting population distributions lead to potential re-arrangement of prey preference and cascading ecosystem effects [43]. These dynamics may be better understood by mapping predation both in the physical landscape and the so-called landscapes of fear, food, disgust and energetics [46–48]. The landscape of fear is the spatial and temporal variation seen in prey movements in response to their perceived risk of predation, typically visualised as peaks and valleys, similarly to terrain maps [49]. For example, in Yellowstone, landscape of fear maps computed for elk were strongly affected by the crepuscular activity patterns of wolves (*Canis lupus*) [50]. Similarly, complex changes in diel activity patterns for roe deer across European landscapes were found in response to the threat of both lynx and humans [51]. These dynamics become more complicated in multi-predator systems where prey must contend with predators using different hunting strategies, resulting in complex landscapes of fear with varying levels of risk [52]. The concept of foodscapes, though developed for herbivores navigating immobile foraging resources [46, 53], can also be extended further up the food chain, as prey resource selection will shape the movements and selected hunting strategies of their predators [54]. The landscape of disgust arises from parasite avoidance behaviour, with further consequences for predator–prey interactions and scavenging decisions [48, 55].

Energetic landscapes, as revealed through accelerometry (i.e., using on-board accelerometer sensors measuring the rate of change of velocity), represent efforts to put animal behaviour and physiology in the context of wider ecosystems and environments [56]. This concept was introduced by Wilson et al. assessing varying movement costs associated with foraging in a heterogeneous environment [57]. Specifically, Wilson et al. [57] compared the foraging dives of imperial cormorants (*Phalacrocorax atriceps*) and the travel costs between the foraging area and the breeding site to a model where individuals were evenly spaced. Complexity was added to the energy landscape definition through (1) cost functions and maps visualising areas of different energetic costs, (2) adding speed and tortuosity of animal movement paths and (3) environmental factors such as wind conditions for aerial travel [58]. More recent considerations have assumed broader energy requirements, to account for thermoregulation and maintenance of body condition, with quantification of individual foraging strategies highlighted as a future direction in using energetic landscapes for population ecology and global change inferences, considering predator performance [59]. Integrating the landscapes of fear and energetics has been discussed elsewhere [56], but there is still little consideration of how species' social systems factor into this picture.

### Social interactions influence predation strategies and may be more flexible than originally described

Research into how sociality affects animal spatial behaviour and general ecology has grown significantly in recent years, as the social landscape, including the distribution and density of conspecifics, can strongly affect the movements and behavioural decisions of individuals [60], (see also: the social resistance hypothesis [61]). Social network analysis in particular is becoming a dominant approach within behavioural ecology [62–65]. As well as looking at interactions within groups, social networks can be used to represent inter-group interactions such as territorial intrusions related to resource abundance [66] and social dynamics of semi-social conspecifics [67]. Investigating the role of species’ social systems, and intraspecific variation in these systems, as a factor influencing energetics requires attention. Conspecifics can affect an individual’s foraging behaviour [68]. For example, information transfer pertaining to foraging sites can occur in colonially-breeding species, such as gannets (*Morus bassanus*) [69]. Social eavesdropping has been reported in vultures, as individuals obtain information about thermals from conspecifics, helping them choose energetically efficient foraging search paths [70]. Social information transmission can influence every stage of predation, encompassing encounter, detection, identification, approach, subjugation and consumption of prey [71].

Whether an animal is social has profound implications for foraging ecology, particularly if social group members cooperate to obtain food, further compounded by dynamic group size responses by prey [72]. Collective hunting allows the takedown of large prey which individual predators could not manage alone [73, 74]. Other species, such as the Ethiopian wolf (*Canis simensis*), target smaller prey individually, even though these predators live in a social group [75]. Some species that typically forage alone or in pairs can opportunistically adapt to cooperative hunting, such as the black backed-jackal (*Canis mesomelas*) [76]. Increasingly, there are reports of cooperative hunting in species thought to only forage alone, including harbour porpoises (*Phocoena phocoena*) [77], goshawks (*Accipiter gentilis*) [78] and yellow-throated martens (*Martes flavigula*) [79]. Where cooperative hunting occurs opportunistically, this may be an attempt by individuals to achieve the benefits of cooperative hunting while minimising the costs which can arise through social foraging. Effort expended during cooperative hunting is not necessarily equal between individuals [80] and how food is shared within a group is influenced by intra-group competition, dominance hierarchies and kleptoparasitism [81, 82]. This opens research avenues focusing on dynamic behavioural decision-making, investigating spontaneous decisions on whether to cooperate to find food, mediated by internal state and animal personality, as well as environmental conditions [83, 84].

### Aims

Here we show how biologging technology can be used to provide new insights in predation energetics. First, we review the development of methods for estimating animal energetics and discuss how more recent technological and conceptual advances facilitate finer-scale, multifaceted insights, primarily through approximation of energy expenditure using accelerometry. Next, we briefly outline the importance of accounting for inter-individual variability. In the subsequent section, we discuss the energetics underlying predation in social and solitary contexts, as hunting alone versus with a team has significant implications for both the intake and output of energy, particularly under changing climate and land use scenarios. We conclude with a section on Future Directions, which suggests methods for optimised experimental design, data collection and analysis, aimed at addressing the questions raised at the beginning of this work (Box 1). Specifically, we posit that growing consideration of energetic landscapes and social networks can be combined. Energetic landscapes effectively capture the influence of abiotic factors on individual movement, behaviour and survival, while social networks often lack due consideration of temporal and spatial scales. We recommend calibrating sensors within captive settings prior to setting up experiments in the wild, which will improve our understanding of shifting animal movement patterns and energetics in the Anthropocene. Further suggestions are made outlining which animals to tag, the study design and which variables to include in statistical models.

## Quantifying predation energetics

### First investigations of animal energetics: from lab to field

Due to the difficulties associated with studying energetics in wild systems, initial investigations into animal energetics were lab-based. Treadmills were, and continue to be, valuable tools in estimating the energetic costs associated with moving at different gaits across multiple species. The use of treadmills to quantify energetics associated with animal locomotion dates back to the nineteenth Century [85] and has expanded to include multiple species across diverse taxa including mammals [86], reptiles [87] and birds [88]. In controlled settings (including laboratories and zoos), treadmills combined with oxygen chambers allow measurement of animal speed and oxygen consumption, allowing energy expenditure to be calculated for many species performing multiple gaits. However, this experimental set-up is not possible with free-ranging wild animals; new developments were required.

The doubly-labelled water method, developed in the 1950s, allows estimation of an animal’s energy expenditure during the window between two blood samples by using isotopically-labelled water to assess carbon dioxide production [89, 90]. With this, research on animal energetics in the wild could commence. It was first used outside the laboratory to assess energy expenditure during rest and flight for homing pigeons (*Columba livia domestica*) [91] and has since been used extensively across diverse wild species [92–95]. While facilitating inferences across diverse systems, the major limitation of this method is the requirement to recapture animals within a rigid timeframe, as the second blood sample must be taken before the isotopes have been eliminated from the body [96]. Additionally, this method provides energetic estimates from the study period as a whole and extensive behavioural observations are required to estimate the costs associated with specific behaviours [96, 97].

### The development of animal-borne sensors

**Fig. 2:**
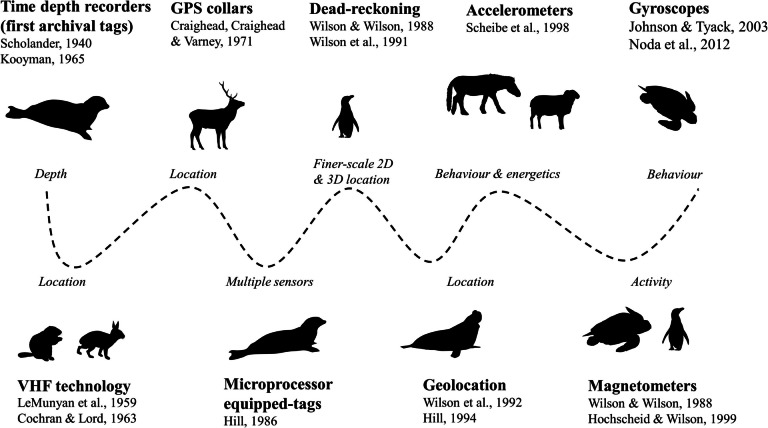
A timeline of key developments facilitating insights into animal energetics. Note all references refer to studies of animal ecology, rather than use of these tools in other fields (e.g., engineering, physics, robotics). Figure references can be found in the reference list as entries [98–101, 106, 122, 125–131]

Time depth recorders, designed to record the diving depths of marine mammals, represented the first use of archival animal-attached sensors [98, 99]. The development of VHF (Very High Frequency) telemetry allowed triangulation of animal location using an antenna to detect pulsed radio signals emitted from an animal-attached transmitter [100–102]. This allowed studies on movements, home ranges and mortality of wild animals to proliferate, and detection of both predator foraging and prey mortality through VHF telemetry continues to provide important insights into predation [103–105]. Satellite collars were first developed in the early seventies [106, 107], allowing location data to be collected and stored at regular intervals via satellite communication. Continued developments expanded options for collecting location information (Fig. 2), and the wide adoption of GPS and Argos satellite telemetry has resulted in large, fine-scale datasets of animal movements across space [108, 109]. Beyond movement trajectories, these data provide detailed insights into behavioural states, including foraging [110, 111]. More recent developments have expanded the range of animal-attached sensors and associated insights, known as biologging (Fig. 2, [112–115]).

### Additional sensors for finer-scale locations and behaviours

Biologging devices incorporating Inertial Measurement Units (IMUs) such as accelerometers (measuring the rate of change of velocity), magnetometers (measuring Earth’s magnetic field, which can be used to give compass-like orientation) and gyroscopes (measuring orientation through angular velocity), allow quantification of fine-scale movement patterns and the relationship between animal behaviour and energetics [116–118]. This is possible as biologging devices allow animal movement to be considered on physiological and biomechanical scales, measuring the individual movements and conditions of the body [119]. As such, these additional sensors provide data distinct from those obtained using even high-resolution locational units (such as those collecting data at the scale of seconds or minutes as opposed to hours).

Using IMUs in tandem with locational units such as GPS allows fine-scale animal behaviour to be mapped in space. This leads to greater insights than achievable with locational sensors alone. Such multi-sensor techniques can advance our understanding of animal energetics with field-based, sub-second-scale measures of movement costs using dynamic body acceleration metrics derived from tri-axial accelerometer data [120]. Deriving energetic landscapes through mapping energy expenditure in space can be used to test optimal foraging theory, by assessing whether animals maximise energy gain while minimising costs as they navigate their environment [57]. Further, precise animal movement paths can be reconstructed in space through dead-reckoning [121]. Dead-reckoning is a path reconstruction method where location data are combined with heading and speed data derived from IMUs [118, 121, 122]. The result is a tortuous, high-resolution path which captures the changes in direction and variable speed of travel undertaken by an animal between subsequent locations. Such highly resolved paths allow more detailed investigations of the precise paths taken by animals and how the costs of moving across different habitat features may shape these.

High resolution GPS and IMU sensors offer different yet complementary information. The behaviour of the species under study and the environment in which it lives dictate the most appropriate sensor choice and sampling regime [123]. Dead-reckoning can be particularly valuable in environments where high frequency GPS sampling is prone to errors or high rates of missed fixes due to habitat composition and/or animal behaviour and posture [123]. High frequency data have been found to provide additional insights into animal behaviour where coarser datasets may result in inaccurate or incomplete interpretations. Some examples include contrasting exploratory movements between bold and shy individuals and detecting multi-animal interactions with consequences for disease transmission [124].

### Detecting foraging behaviour

Information about the type and amount of food ingested by animals can answer fundamental ecological questions relating to how animals manage their energy budgets in the wild [132]. Inter-mandibular angle sensors (IMASEN), placed on animal jaws, have been used to reliably determine prey ingestion [133]. More commonly, fine-scale movement data are used to reconstruct predation events. Clusters of GPS locations may indicate kill sites, often with field visits for verification [111, 134]. It should be noted that this method is biased towards large predators hunting large prey, with kill sites of small prey typically classified at lower accuracies [134]. Hidden Markov models (HMMs) allow movement data to be categorised into discrete states [135]. Although these states are typically not verified behaviours, kill sites can also be used to confirm HMM-defined predation occurrences [136].

**Fig. 3:**
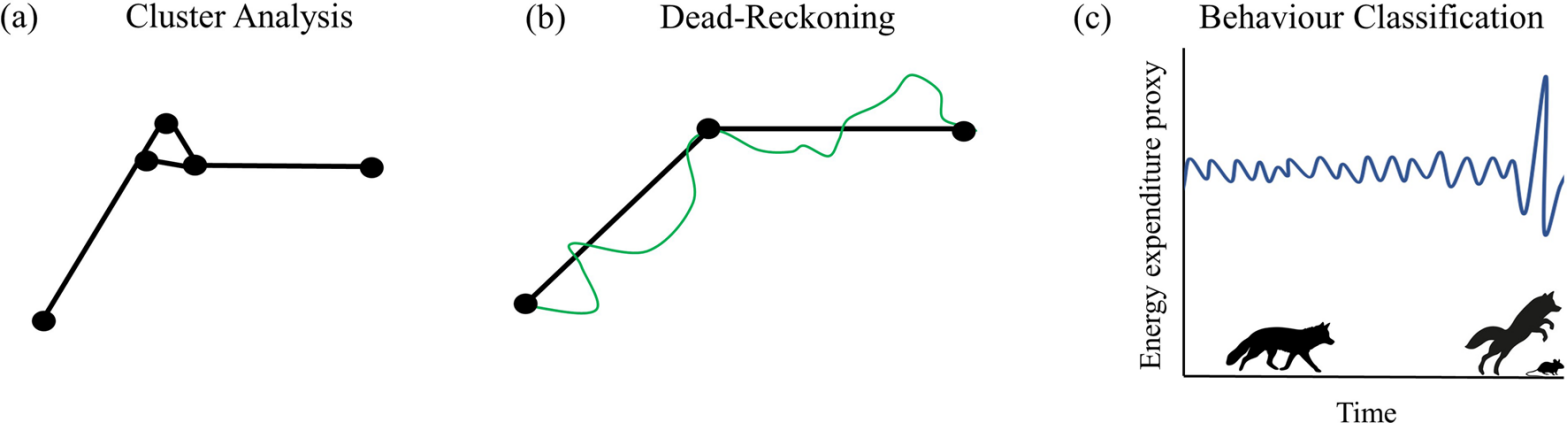
Examples of analysis methods for GPS and Inertial Measurement Unit data with relevance to predation energetics. **a** Cluster analysis of GPS data allows detection of kill sites by detecting spatially and temporally clustered locations, indicated by the dots here, e.g. [134, 144]. **b** Dead-reckoning animal movement paths using GPS, accelerometer and magnetometer data allow the tortuosity of movement paths to be captured and can be used to reconstruct paths of hunting predators [121]. Here the black line represents the straight-line distances between subsequent GPS points, while the green line represents a dead-reckoned path. **c** Behaviour classification of data from IMU sensors such as accelerometers can be used to distinguish predation from other behaviours e.g., [145]. Note that the proxy for energy expenditure here can take the form of raw sensor data such as individual acceleration axes or metrics such as VeDBA or ODBA

Foraging strategies vary depending on the food items targeted, habitat type and whether foraging is cooperative or solitary [79, 137–139]. As different hunting strategies involve different body postures and energetic signatures, it should be possible to extract these separate hunting strategies from biologging data (Table 1). For example, combined tri-axial accelerometer and GPS data have shown promise in elucidating the energetics underlying prey capture by large predators like African leopards (*Panthera pardus*) [140] and high frequency acceleration data have been used to classify behaviours related to foraging in smaller predators such as the Arctic fox (*Vulpes lagopus*) [141]. As speed estimates can be derived from both GPS and acceleration data, and magnetometers can capture the tortuosity of animal movement paths [142], these technologies present opportunities to look at speed, pursuit and evasion in hunting predators and fleeing prey (Fig. 3; [143]).

**Table 1:** A list of key sensors linked to behavioural interpretations relevant to predation energetics

Sensor	Behavioural inferences	Examples
GPS units	Identify locations visited during foraging trips	[146]
	Calculate distances travelled	[147]
	Identify kill site clusters	[144]
Accelerometers	Identify postures and movements related to pursuing prey	[148, 149]
	Quantify predation success rate	[149]
	Turns taken during foraging trips	[143]
Magnetometers	Identify postures related to foraging	[150]
	Turns taken during foraging trips	[151]
	Dead-reckoning	[121]
Proximity sensors	Social interactions	[152]
	Social foraging	[153]
Intermandibular Angle Sensor (IMASEN)	Opening/closing mandible during foraging	[154]
Camera	Direct footage of all predation-related behaviours	[28]
Microphone	Recordings of prey cries	[155]
	Recordings of chewing sounds	[156]
	Detection of calls associated with foraging	[156]

### Linking predation theory and biomechanics to sensors

Certain aspects of hunting behaviour should be kept in mind when quantifying predation energetics through biologging given the physiological and biomechanical insights available through such sensors. For example, prey may undertake complex escape manoeuvres as they choose where to flee and predators follow this route. As such, turning dynamics of coursing predators during a chase have been shown to vary with prey species and the mass of both predator and prey [143]. Combining movement data with high resolution habitat data, i.e., those collected using remote sensing and LiDAR methodologies, e.g., [157], represents the highest accuracy framework for assessing turning dynamics in predator–prey chases (see Future Directions). Both pursuit and evasion have important energetic consequences, which ultimately determine hunt outcomes [143, 158].

**Fig. 4:**
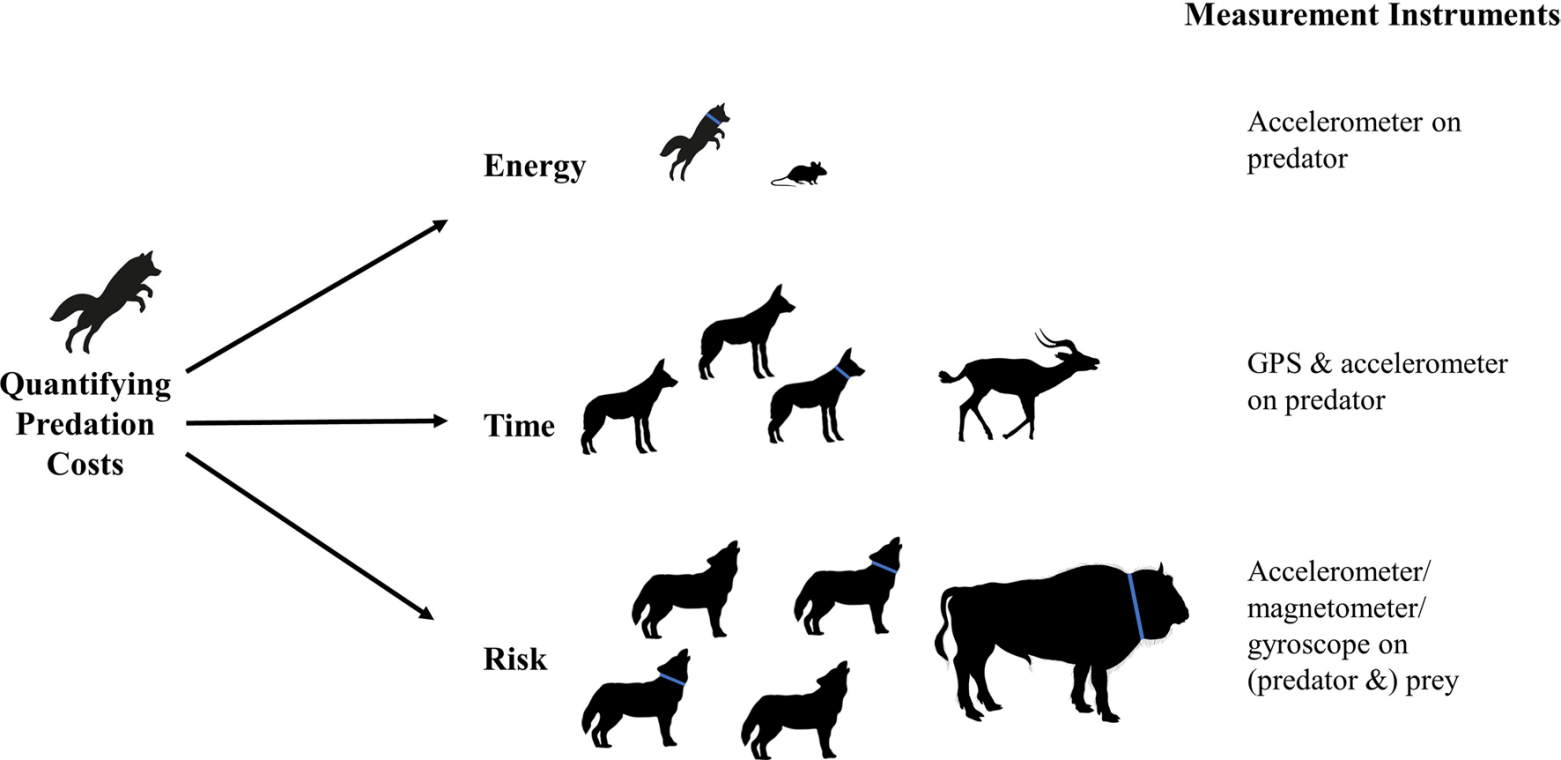
Predation requires investment of energy and time, while involving significant risks associated with attacking and subduing prey. Example predator–prey pairs are shown here, with predation costs linked to sensors which can be used to quantify them. Tagged animals are indicated with blue collars on the relevant silhouettes. Accelerometers allow the calculation of Dynamic Body Acceleration proxies which provide estimates of energy expenditure which can be matched to distinct behavioural states. GPS and accelerometer data allow the start and end points of predation to be identified so that time spent hunting can be quantified. Inertial Measurement Units can be used to assess animal posture, to detect defensive or aggressive behaviours exhibited by prey and alert postures to be detected in predators. Predator retreat may also be identifiable from dead-reckoned movement paths

The energetic costs of predation can be split into costs of (1) locating prey, (2) pursuit or ambush and (3) restraining and killing the prey, respectively. More time spent on any of these aspects results in higher energy expenditure, but the costs of each step are unequal and vary between predator–prey dyads. The energy required for separate stages of predation can be estimated through the collection of biologging data (Fig. 4). For example, locating prey is less costly than the pursuit per unit time, where terrestrial predators switch from walking or trotting search gaits to running pursuit gaits [159]. Behaviour classification of movement modes can identify and assign approximate costs to such behaviours. Longer search times could involve finding easier prey with shorter pursuit and restraint times [160]. Therefore, time and energy are separate costs, but time spent on a given activity is critical to the total energetic cost of the hunt. Costs incurred by previous unsuccessful hunts and the sum of other behaviours performed by the animal should also be taken into consideration. Failed hunts, scavenging and foraging for smaller food items may also be accounted for through data from accelerometers and other IMU sensors as behaviour classification methods continue to advance [141, 145]. Developing classification methods for these complex behaviours may be assisted considerably by the increasing use of animal-attached cameras and microphones, allowing further verification of IMU sensor outputs [155, 161]. Proximity sensors can be used to detect cooperative foraging in predators [153], as well as encounters between predators and prey [162].

As well as facilitating fine-scale, behavioural insights, animal-attached technology can also provide important information on broader ecological scales. Understanding the energetics of predation can provide information on trophic cascades and predator–prey dynamics with consequences for whole ecosystems [163]. Integrating biologging data into Dynamic Energy Budget-Individual Population Models (DEB-IPMs) has been identified as a powerful emerging method to link individual level behavioural energetic trade-offs and metabolic processes to population dynamics including survival and reproduction, with due consideration to environmental change [164]. As such, despite the fine-scale nature of biologging data and often short deployment periods, these data can provide important, broader-scale inferences for population ecology [165, 166].

## Inter-individual variability

There has been relatively little consideration of how consistent inter-individual differences (i.e., animal temperament or animal personality) might affect hunting prowess. Individual variation may lead to specialisation in solitary hunters like octopuses [167] or distinct roles in cooperative hunters, as seen in harbour porpoises [77]. Further, predator and prey personalities may interact in feedback loops [84], with some empirical evidence suggesting both predator and prey personality may interact with consequences for predation attempt outcomes [168].

If individuals adopt flexible foraging strategies such as exhibiting prey preference based on prey size and availability, as well as the broader ecological context, then it is reasonable to assume that differences in strategy will arise between individuals. Some differences may be linked to factors such as age and sex [9], though further variation may be attributed to consistent intra-individual variability. This can be measured by considering the repeatability and predictability of behaviours. Protocols for extracting measures of personality from biologging data have recently been developed and are growing in popularity [169]. To date, these methods have largely focused on using parameters extracted from GPS data, including distance moved and activity patterns [170] though there is considerable scope for IMU sensors to yield additional insights into individual variation in activity level and space use as influenced by foraging [171, 172]. Individual variation in activity rhythms and how prey are approached and hunted may affect predation strategies and the roles performed by cooperative hunters, with potential energetic implications. These patterns can be better understood by using movement metrics such as daily travel distances and the amount and timing of activity to detect consistent behavioural differences between individuals (for full review, see [169]). Despite the growing attention given to understanding animal personality in ecology and evolution [83], the role of personality in driving predator behaviour is still far from understood.

## Social predators

An animal’s social environment can affect both the costs and benefits associated with finding food, warranting specific considerations for social predators. Social foraging can decrease the time and energy an individual invests in locating and consuming prey [146, 173] and enable access to prey which cannot be obtained by a single predator [74, 174]. Unequal effort invested in securing prey and gained through how food is shared can present new challenges, however [5, 81]. Whether a predator hunts alone or with a team therefore has implications for how animal-attached sensor data should be interpreted and which wider conclusions on predation energetics can be drawn. In this section, we provide a brief overview of challenges and considerations for studies on social predators.

Often when studying social species, tags are deployed on one or a few members of multiple social groups, to gain insights into the larger population, though with consequences for our understanding of social group interactions [175]. One of the primary difficulties in interpreting tag data from social foragers is that both the energy expended in acquiring a meal and the energy intake from successful predation may be unequal between group members. This is particularly true where group members perform different roles during a hunt [176, 177]. This makes it difficult to extrapolate energy intake and output from tagged individuals to other group members, and indeed conspecifics more generally. This is particularly complex where social group sizes are unknown or fission–fusion dynamics are at play, leading to variable numbers of predators present at each predation event. The percentage of a social group or population which has been tagged affects how readily social interactions can be detected. Detection of interactions between members of the social group is further influenced by sampling frequency, which must also be taken into consideration when studying group dynamics [175]. The strengths of within-group and between-group social interactions may also vary depending on ecological conditions, e.g., in lions [178]. Thus, an additional complication is estimating the distribution of conspecifics across the landscape. Additional data, such as sightings, combined with tag data, may be used to build a social landscape providing the likely density of conspecifics from different groups [60]. This will likely require intensive sampling and surveying across potentially large areas. Further analysis considerations are required for behaviour classification of IMU sensor data. For example, when some but not all members of a social group have made a kill and an untagged individual does not participate in the hunt but feeds on said kill, it may not be possible to decipher whether this feeding instance represents active predation by the group or opportunistic carcass scavenging.

Studies of predation energetics should consider the range of prey species taken by a social predator, as the degree of cooperation may vary with prey size and relative risk to the predator. This is particularly relevant to generalist predators with wide distributions, the range of which may encompass different habitat types and prey species compositions. This is not static, for example larger wolf packs are more cooperative during a hunt when hunting more dangerous prey [74]. Where possible, simultaneous tagging of predators and their prey can improve our knowledge of interactions between groups of predators and dangerous prey (Fig. 4).

It is important to note that other factors affect the size of animal social groups, including defending vulnerable young and territories. This may explain why social groups are often larger than identified optimum group sizes for cooperative hunting [179, 180] and why some species, like the Ethiopian wolf, occupy shared territories and breed cooperatively but forage alone [75]. Even when the hunt itself is cooperative, feeding may still be competitive when groups contain more individuals than are necessary for optimised cooperative hunting [81, 82]. Dominant individuals may limit food access to more subordinate group members [181], though other factors beyond social hierarchies can also affect the roles social group members perform in hunts and the related energy intake and output from a kill. Further studies involving tagging all or most individuals within a social group can shed light on these cooperative hunting dynamics. While this is not practical in all cases, even studies on a single social group can help address these knowledge gaps and aid data interpretation where few or sole individuals in a group have been tagged [182].

## Future directions

In this review, we have summarised key theory in the predation energetics literature, outlined the development of biologging tools for measuring animal energetics and highlighted key considerations which must be accounted for when investigating intra-individual variability or working with social predators. We conclude by proposing future directions in predation energetics research, which will be key in identifying different energetic costs and gains experienced by predators in a changing world.

**Fig. 5:**
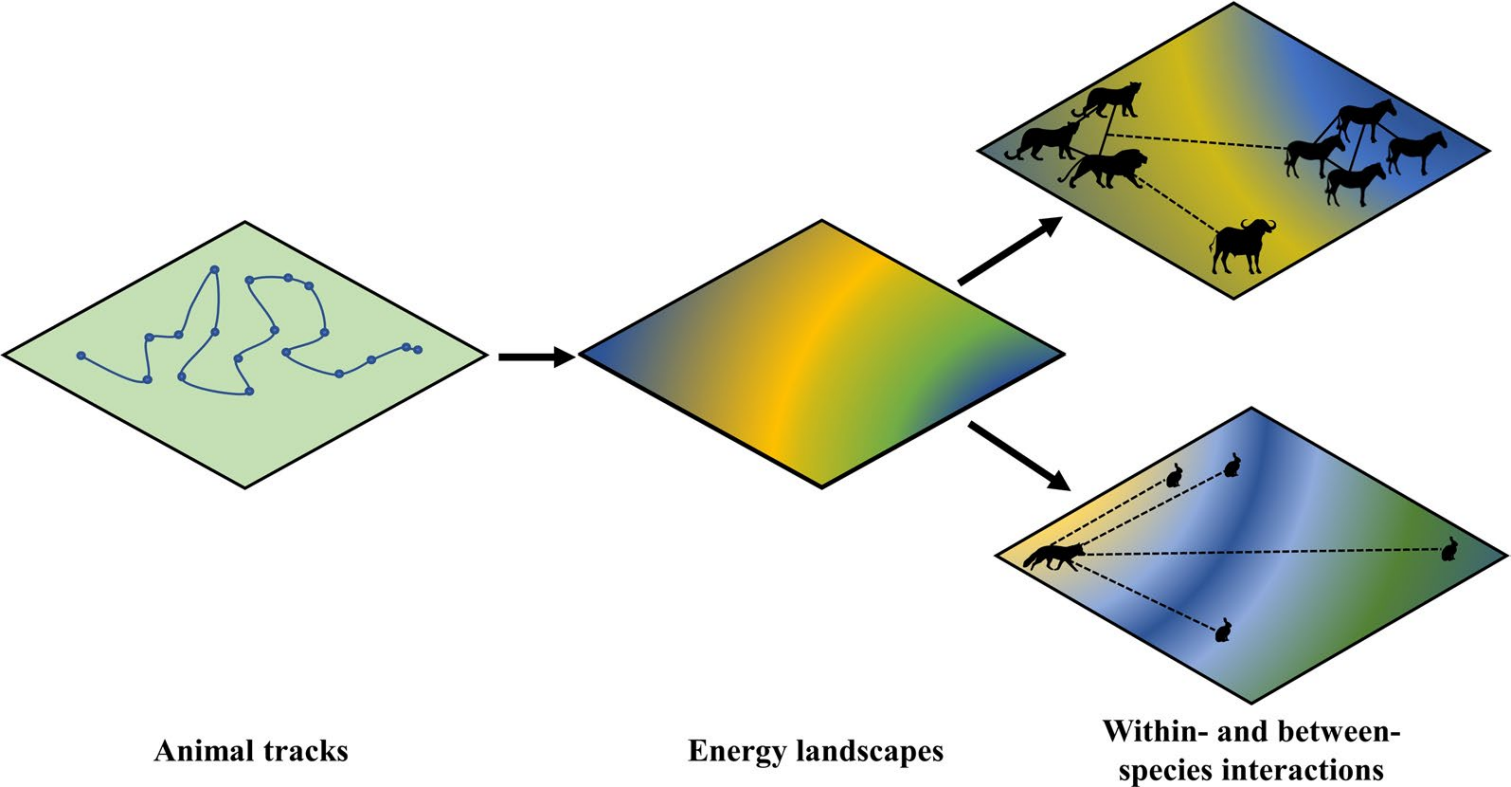
Infographic contrasting the energetic costs and gains between a social group of cooperative hunters and a solitary predator, incorporating energetic landscape and social network concepts. Sensor data on animal location and energetics can be computed into energy landscapes, which can in turn influence prey selection and encounter rates. The colour gradients here represent hypothetical energetic landscapes, where movement costs vary across the habitat in question. Solid black lines indicate interactions between social conspecifics, while dashed lines indicate directions of interest to predators due to prey presence

1. *Integrating energetic landscapes and social networks*. Animals navigate a spatial landscape and other animals, including predators and prey, affect movement and energetics in a similar fashion to abiotic landscape factors. These biotic factors have unequal avoidance and attraction effects with consequences for how animals navigate their environment [185]. Attraction to or avoidance of conspecifics and/or heterospecifics may result in suboptimal use of the physical landscape (e.g., expending more energy to traverse through rough terrain to search for prey or avoid competition). Conversely, an animal may choose the least costly path to navigate the local terrain, which then affects its biotic interactions. Studying such interactions within ecological communities is increasingly feasible due to large-scale tracking initiatives such as ICARUS [186] and data-sharing platforms such as Movebank (which also contributes a data standardisation philosophy; [187]). Energetic landscapes, which consider the costs of navigating the physical landscape, and social networks, which define the relative strength of social interactions, can be unified to consider the abiotic and biotic factors shaping animal movement patterns in tandem. For example, integrating these methods could be used across predator and prey communities to investigate how the physical environment influences prey selection. Further, thermal shelters are likely to become more important to many species under climate change [188, 189], which may have knock-on effects for prey detection and predator avoidance strategies.
  Social network analysis (in both intra- and inter-specific systems) offers an analytical means of assessing the role of social interactions in species ecology [64]. Social networks are typically visualised as nodes clustered by interaction frequency, but can be overlaid onto maps to better assess the role of spatial proximity and environmental variables in determining association strength, e.g., [67]. This process can be taken a step further by overlaying social networks onto mapped energy landscapes, where individuals have been tagged with locational units and accelerometers (Fig. 5). Beyond visualisation, the recently developed R package *aniSNA* can be used to resolve autocorrelation issues encountered in the computation of social network metrics using GPS data [188]. The most robust social network metrics for a given dataset, determined with due consideration to sampling regime and sociality, can then be modelled with measures of energy expenditure derived from mechanistically modelled energetic landscapes, integrating data on species interactions and energy expenditure. This unleashes new opportunities to test specific hypotheses on how social-energetic landscapes vary as a function of, for instance, prey availability or environmental conditions such as temperature, or how individuals modify the strength of their interactions with different prey species across habitats which are more or less costly to traverse. Energetic landscapes under global change scenarios (e.g., [59]) can be adapted to include shifting predator and prey interaction patterns, represented through social network centrality metrics such as mean network strength, to quantify altered ecosystem dynamics. Considering energetic landscapes in conjunction with within- and between-species interactions may expand proposed spatial-social data concepts [189] to provide new insights on how other animals affect how an individual navigates its environment.

2. *Refining data collection and analysis procedures using captive and domestic animals*. Pilot studies on captive and domestic animals allow refinements before wild tag deployments. Zoos provide settings where sensor data can easily be verified for improved data analysis procedures ahead of wild deployments [172]. Captive studies can also have welfare benefits by piloting device attachment and deployment methods. Such studies can also detect potential species-specific considerations required ahead of long-term field deployments e.g., maned wolves (*Chrysocyon brachyurus*); English et al., unpublished data. While there are limitations to using surrogates [190], with careful interpretation, data from captive and domestic animals can improve behaviour classification procedures for biologging data [191]. This can be particularly useful when investigating complex postures and motions such as those associated with feeding.
3. *Tagging multiple or all individuals in a social group*. Simultaneously tagging multiple or all individuals in a single social group is rarely done for multiple reasons. Most studies typically have limited numbers of tags and aim to spread them across multiple social units so that broader population insights can be gained [175]. Deploying tags in discrete social groups can also address statistical assumptions of independence of data points, depending on the analysis methods used. These constraints are valid, but currently limit our fine-scale knowledge of within-group interactions, including distinct roles which may be performed during coordinated hunting behaviour. Studies which target an entire social group can reveal whether a hunt is truly cooperative and quantify the influence of habitat on pursuit predation, with important considerations for how focal species may adapt in changing land use and climate scenarios [23]. While tackling entire social groups is easier where groups are small, it is becoming increasingly feasible and common to also tag larger social groups (e.g., [192]). While tagging multiple or all members of a social group will lead to advances in our understanding of animal societies, tag burden should be kept in mind and research questions should be well formulated to ensure maximum information gain from studies with potential higher overall tag burden. Researchers can also implement non-invasive technologies to collect empirical data on group size, such as camera traps and drones, for example in scenarios where tagging all members of a social group is not feasible due to economic or logistical constraints, or to verify social bonds where these cannot easily be ascertained by an observer. For example, camera traps have been used to detect high contact rates between red foxes (*Vulpes vulpes*), which are considered solitary foragers, where food availability is high [193].
4. *Taking social groups as individual units to compare inter-group communication and interactions.* Complementary to studies of within-group interactions, there is scope for further consideration of between-group interactions, where territoriality may (at least occasionally) be weaker than first described, as has been found in black-backed jackals [76]. This also applies to solitary species which may interact socially with conspecifics in neighbouring territories more readily than previously thought (e.g., maned wolves [194]). These interactions may be aggressive or affiliative and include communication through scent-marking and vocalisations. These forms of communication also shape how an animal perceives and therefore navigates its environment, with consequences for territoriality and therefore the resources available to the territory holder. Such interactions are more difficult to visualise and frame in a social-energetic landscape context, but mapping instances of scent-marking behaviour classified through IMU sensor data [195] and continued advancements in acoustic recording research [196] may improve our understanding of these non-visual communication channels in shaping how animals move through their environment.
5. *Simultaneous tagging of predators and their prey.* As well as investigating the within- and between-group interactions of predators, further studies with simultaneous tagging of both predators and their prey are required. Studies where members of a predator and prey species within the same study area are tagged with location sensors can provide valuable information on predator and prey activity rhythms, their degree of spatial overlap and how these may interact with landscapes of fear and energetics [197, 198]. Where possible, tagging predators and prey simultaneously with IMU sensors may provide detailed data on individual hunt dynamics. Such data can be used to characterise chase paths, turning dynamics and evasive movements [158]. While the likelihood of a tagged predator hunting tagged prey is still quite small in most systems, any instances where this is recorded is likely to have profound insights into how the pursuit and restraint techniques of the predator and the escape strategies of the prey interact with one another. As well as these tagging approaches, continued advancements in tracking animal locations and postures through drone-collected aerial imagery [199] may hold significant potential in capturing pursuit and evasion dynamics of predators and prey.
6. *Account for factors such as hunting success rate and relative prey energy value in statistical model structures*. Fine-scale biologging data and related behaviour classification can also contribute additional variables to include in models of predation energetics. For example, where hunting can be defined, the approximate energetic costs of distinct prey species and their energy value when obtained (either estimated from time spent feeding if clear from IMU sensor traces or through a proxy derived from prey body size or estimated caloric value) can be included in model structures. Models explaining the likelihood of successful predation of a given prey would benefit from including the approximate energy value of the prey, encounter rate, handling time and individuality. Conversely, failed predation attempts can be an important consideration when considering a more general model of a predator’s energetic balance.
7. *Increasing the diversity of species tagged and included in such studies*. One of the limitations of animal-attached sensors is that tag size and weight can limit the potential for the use of such technology on smaller animals. Considerable advancements have been and continue to be made, however, such as biologger sensor networks developed for tracking bats [200]. Though the development of smaller tags facilitates deployments on smaller species, these developments should also aim to facilitate the use of reduced mass tags on individuals to minimise potential deleterious effects [201]. Biologging studies are also biased towards mammals, and to a lesser extent fish and birds [202]; efforts should be made to increase the diversity of species represented in such studies.
8. *Sampling designs tackling the influence of climate and habitat modification on foraging behaviour*. Predator–prey interactions are key to trophic ecology and it is therefore important to assess energy balance in these relationships in a changing world. Further, robust understanding of energy intake and output is required to understand species responses to climate and habitat change. These questions can be tackled, for example, by comparing energetics across populations with different weather patterns to approximate species responses to climatic shifts [37]. Studies on wildlife in human-dominated landscapes such as urban areas or agricultural land can yield insights for areas undergoing current land use change*.*

The future directions presented here offer a roadmap to further expand our knowledge of predation energetics using animal-attached sensors, accounting for sociality, individual variation and global change. Advances in animal-attached tagging technology have rapidly expanded the ecologist’s toolkit for understanding animal energetics. The tools presented here, coupled with thoughtful study designs and integrated analysis concepts, can facilitate substantial advances in our understanding of predation energetics in a changing world.

## Data Availability

Not applicable.
